# Cognitive Reserve in Parkinson's Disease: The Effects of Welsh-English Bilingualism on Executive Function

**DOI:** 10.1155/2015/943572

**Published:** 2015-04-01

**Authors:** John V. Hindle, Pamela A. Martin-Forbes, Alexandra J. M. Bastable, Kirstie L. Pye, Anthony Martyr, Christopher J. Whitaker, Fergus I. M. Craik, Ellen Bialystok, Enlli M. Thomas, Virginia C. Mueller Gathercole, Linda Clare

**Affiliations:** ^1^Bangor University, Gwynedd, Bangor LL57 2DG, UK; ^2^Betsi Cadwaladr University Health Board, Llandudno Hospital, Conwy LL30 1LB, UK; ^3^NISCHR CRC North Wales Research Network, Bangor University, Gwynedd, Bangor LL57 2PZ, UK; ^4^University of Exeter, Devon, Exeter EX4 4QG, UK; ^5^Rotman Research Institute, Toronto, ON, Canada M6A 2E1; ^6^York University, Toronto, ON, Canada M3J 1P3; ^7^Florida International University, Miami, FL 33199, USA

## Abstract

*Objective*. Bilingualism has been shown to benefit executive function (EF) and delay the onset of Alzheimer's disease. This study aims at examining whether a bilingual advantage applies to EF in Parkinson's disease (PD). *Method*. In a cross-sectional outpatient cohort of monolingual English (*n* = 57) and bilingual Welsh/English (*n* = 46) speakers with PD we evaluated the effects of bilingualism compared with monolingualism on performance on EF tasks. In bilinguals we also assessed the effects of the degree of daily usage of each language and the degree of bilingualism. *Results*. Monolinguals showed an advantage in performance of language tests. There were no differences in performance of EF tests in monolinguals and bilinguals. Those who used Welsh less in daily life had better performance on one test of English vocabulary. The degree of bilingualism correlated with one test of nonverbal reasoning and one of working memory but with no other tests of EF. *Discussion*. The reasons why the expected benefit in EF in Welsh-English bilinguals with PD was not found require further study. Future studies in PD should include other language pairs, analysis of the effects of the degree of bilingualism, and longitudinal analysis of cognitive decline or dementia together with structural or functional neuroimaging.

## 1. Introduction

The theory of cognitive reserve has been proposed to explain the mismatch between the degree of brain pathological changes and observable clinical manifestations [1], in relation to the development of dementias such as Alzheimer's disease (AD) [2], and to account for interindividual rates of cognitive decline. There has been recent interest in the effects of cognitive reserve in Parkinson's disease (PD) [3, 4]. Bilingualism is a form of cognitive reserve which may delay the onset of AD [5, 6] but it has not been studied in PD.

PD is an age-related neurodegenerative condition which is associated with cognitive impairment. Up to 25% of newly diagnosed people with PD who do not have dementia and up to 90% of people with PD at any stage experience mild cognitive impairment (MCI), with an increased risk of developing PD dementia (PDD) [7]. Cognitive impairment in PD is manifested particularly in abnormalities of executive function (EF) [8], visuospatial function, attention, and memory. Impairment of EF in PD may, however, influence adversely quality of life, health status, and carer burden [9] and reduce awareness of functional limitations [10].

Psychosocial factors, including lifelong level of cognitive, social and physical activity, and the size and complexity of social networks, are known to contribute to delaying the onset of cognitive disability in later life through the promotion of cognitive reserve [11–13]. The cognitive reserve hypothesis states that individual differences in the processing of tasks provide varying levels of reserve against brain pathology [2]. Childhood intelligence, higher education, and level of occupation contribute to cognitive reserve, which may influence cognitive ageing and decline [2, 14]. A systematic review has confirmed that higher reserve is associated with less cognitive decline with age [15]. As cognitive reserve cannot be measured directly, all studies use proxies with education, intelligence, occupation, engagement in leisure activities, and engagement in cognitive activities being the ones employed most commonly [1, 2, 16]. Proxies used less commonly include height, head and brain size [17], and bilingualism [6]. Cognitive reserve is seen as serving a general protective function and is not specifically associated with any given disease or condition. Therefore, the effects of enhanced cognitive reserve should be evident both in healthy ageing and in a range of neurodegenerative conditions. Previous reviews have proposed a role for cognitive reserve in PD [3, 4, 18]. Cognitive reserve, based on the proxy of education, has been shown to enhance performance of many cognitive tests in PD in cross-sectional studies and slow the progression of global cognitive decline [3, 18] with one study of intelligence also showing a positive effect [19].

Lifelong bilingualism has been shown to delay the onset of AD, with this bilingual advantage being attributed to cognitive reserve [6]. Another longitudinal study found the number of languages spoken contributed to the prediction of cognitive test scores beyond the effect of other demographic variables [21]. Two other longitudinal studies however did not confirm a bilingual advantage [22, 23]. Bilingual patients diagnosed with probable AD exhibit substantially greater amounts of brain atrophy than monolinguals [24] protecting against the cognitive effects of age-related declines in white matter integrity [25]. The degree of bilingualism may be important in promoting cognitive reserve [26], and the nature of the particular languages may affect the nature of the interaction with neuropsychological function and bilingualism [27]. One study has shown an advantage for multilingualism [28] but not for bilingualism on age of AD diagnosis, whereas other studies have not shown the effect of language at all [22, 29]. Bilingualism has been shown to enhance cognitive control, conferring an advantage in EF [30]. This bilingual advantage has been shown in studies of MCI, where the effect may be specific to single-domain amnestic MCI, which has been linked with progression to AD [31]. Importantly, multiple-domain amnestic MCI that included EF impairments showed no protection from bilingualism, an effect the authors explain in terms of the loss of EF as a potential compensatory system. A recent prospective study showed that while bilinguals may have better executive function and memory at baseline there was no independent association between bilingualism and cognitive decline or dementia [32]. If the bilingual advantage is mediated through changes in EF, the effect may be best explored in conditions such as PD where EF may be compromised [9].

A number of outstanding questions about the nature of the bilingual advantage have been identified [30]. Two important questions relate to this current study. What are the limits and boundary conditions for the bilingual advantage and why are bilingual advantages not always found? What is the role of the standard components of executive control—inhibition, attention switching, and working memory—in bilingual differences in processing? A better understanding of the cognitive and neural mechanisms underlying the effects and limitations of the bilingual advantage will provide valuable information about protective and preventive factors that can mitigate cognitive impairment and dementia in conditions like PD in which EF may be compromised.

This study aims at examining the effects of bilingualism on EF in age-related neurodegenerative conditions by comparing the performance on tests of EF of bilingual and monolingual individuals with PD. The main hypotheses were firstly that bilinguals with PD will show better performance on executive control tasks than monolinguals and secondly that bilinguals with PD who have a higher degree of bilingualism will show better performance on executive control tasks than those with a lower degree of bilingualism.

## 2. Method

### 2.1. Design

This was a cross-sectional outpatient cohort study which compared bilingual Welsh/English and monolingual English speakers with PD. This was part of a larger study, the Bilingualism as a Protective Factor in Age-Related Neurodegenerative Conditions (BANC) study, which examined the effects of bilingualism in people with PD and AD and healthy older people. The study received ethical approval from local National Health Service and University ethics committees and complied with the Declaration of Helsinki.

### 2.2. Participants

Patients diagnosed by a Movement Disorder Specialist as having PD according to UK Parkinson's Disease Society Brain Bank criteria [33] were recruited through Movement Disorders Clinics in North Wales. Wales is an officially bilingual constituent nation of the United Kingdom, with the counties of North Wales having above average proportions of Welsh speakers, with the highest proportions in western areas as follows: prevalence (from west to east) is 63% in Anglesey, 65% in Gwynedd, 35% in Conwy, 31% in Denbighshire, 17% in Flintshire, and 18% in Wrexham [34, 35]. Welsh has a completely different derivation and structure from English with Welsh being a Brythonic Celtic language and English being of Germanic origin. Welsh contains a number of vocal sounds not present in English and has a very different grammar structure. Target recruitment was 50 bilingual Welsh/English speakers and 50 monolingual English speakers from across North Wales. “Bilingual” was defined as speaking both languages for all or most of one's life and being fluent in both languages which were both used on a daily basis. It was predicted that, with a sample size of 50, 80% power would be achieved when comparing monolinguals and bilinguals for detecting an effect size of 0.55 when the correlation of the covariates with executive control is 0.3. Exclusion criteria were MMSE < 18, bilingualism in other language pairs except Welsh/English, other significant neurological disease and lack of ability to provide informed consent.

All tests were performed in the motor “on” state as defined by the participants. All tests were performed in English unless otherwise stated.

### 2.3. Measures

#### 2.3.1. Baseline Demographics and Illness Severity

A structured interview was conducted to obtain demographic information including age, socioeconomic status (SES) [36], educational level in years and achievement level, lifestyle, and time since PD diagnosis. PD motor severity was measured by the Unified Parkinson's Disease Rating Scale part 3 motor score (UPDRS) [37] and Hoehn and Yahr severity (H&Y) [20]. The levodopa equivalent dose (LED) of antiparkinsonian medications was calculated for each participant [38]. The burden of cognitive anticholinergic side-effects of medication was calculated using the anticholinergic cognitive burden scale, where a score of 3 or more is regarded as a significantly high burden [39]. Mood was assessed with the Hospital Anxiety and Depression Scale (HADS) [40]. Global cognitive screening was done using the Mini-Mental State Examination (MMSE) [41].

#### 2.3.2. Language Tests

Further baseline descriptive tests were tests of English language ability. These were the National Adult Reading Test-Revised (NART-R) [42], the British Picture Vocabulary Scale (BPVS; 43), and the Boston Naming Test (BNT) [43], with the latter being conducted in both English and Welsh in bilinguals. Language status was assessed using a short language questionnaire which assessed the frequency, setting, and fluency of use of languages [44].

#### 2.3.3. Executive Function and Sustained Attention

Executive tests were selected to cover each subdomain of EF. Mental generativity and speed were assessed with the Design Fluency and Verbal Fluency, letter fluency and category fluency, subtests from the Delis-Kaplan Executive Function System (D-KEFS) [45] and with Raven's Coloured Progressive Matrices (RCPM) [46] which is a test of nonverbal reasoning. In verbal fluency and category fluency tests in English, where bilingual individuals had significant Welsh intrusions (more than one or two) or reverted to Welsh, the results were excluded (these were the only tests where Welsh intrusions occurred). Working memory was assessed with the Wechsler Memory Scale, backwards Spatial Span and backwards Digit Span subtests [47], and the Keep Track task [48, 49]. Inhibition and management of response conflict were assessed with the Test of Everyday Attention (TEA) Elevator Counting with Distraction subtest [50], computerised Simon task [51, 52], computerised Stroop colour word naming [51, 53], and computerised Go No-Go task [54]. Set shifting and switching were assessed with D-KEFS Trail Making Part 4 subtest [45]. Sustained attention was assessed with the TEA Elevator Counting subtest [50].

### 2.4. Planned Analyses

Possible covariates among the demographic variables (age, SES, education, time since diagnosis, measures of illness severity, and measures of mood) and clinical background variables (H&Y stage, UPDRS, time since diagnosis, age at diagnosis, MMSE, HADS depression, and HADS anxiety) were sought. Chi squared tests were used for categorical variables and ANOVA for quantitative variables to assess the relationship between the outcome variables and language grouping (monolingual versus bilingual).

Comparing monolinguals and bilinguals, categorisation of participants according to educational level indicated that monolinguals were significantly better educated. Because the educational level variable used was categorical, it was included in the analysis as a fixed factor rather than a covariate. Educational level was added followed by language (monolingual, ML, or bilingual, BL) into a two-factor ANOVA without the interaction term, using the type I (regression) sum of squares. This analysis was used to determine whether or not language had an effect on the outcome variables after accounting for any effect of educational level. Comparisons are based on cases with valid available data and no imputation of missing data was performed.

Effect sizes were calculated as the difference between the monolingual and bilingual mean values divided by the square root of the error mean square from the ANOVA table. This provides the standardised mean difference (SMD) after accounting for the effect of educational level. Confidence intervals were calculated using the method described by Hedges and Olkin [55].

Cluster analysis based on average between-group linkage using squared Euclidean distance was carried out on responses to the language use questionnaire in order to categorise the bilinguals depending on the degree of everyday use of two languages. A two-cluster solution was found to have the best fit, with Cluster 1 representing less frequent Welsh speakers and Cluster 2 representing more frequent Welsh speakers. Comparison of the two clusters on baseline demographic and clinical variables using Chi squared tests for categorical variables and ANOVA for continuous variables yielded no significant differences and hence no identifiable covariates; therefore, a one-way ANOVA was used to compare the two clusters on the outcome variables.

The procedure outlined by Gollan et al. [26], in which bilingual index scores were calculated using two language versions of the BNT (here Welsh and English) by dividing the proportion of pictures named correctly in the language which produced a lower naming score by the proportion named correctly in the language which produced the higher naming score, was also carried out. The results were then correlated with outcome variables using Spearman's correlation due to the skewed nature of the bilingualism index scores.

The Holm-Bonferroni correction [56] for multiple comparisons was applied separately for descriptive neuropsychological variables and outcome measures for the ANOVA results but not correlations.

All statistical analyses were performed using SPSS v20 (IBM Corporation, NY, USA).

## 3. Results

### 3.1. Demographic and Disease Variables

57 monolingual English and 46 bilingual Welsh/English speakers with PD were recruited. 12 of the monolingual group had learnt some Welsh (2 from age 5, 2 from age 11, and 8 in adulthood) but did not fulfil the definition of bilingualism used in the study. One of the adult Welsh learners (learnt at age 36) did speak Welsh at home but less than 50% of the time, 4 used Welsh only very occasionally, and the others used no Welsh. Two of the monolingual group participants who learnt Welsh in adulthood were also proficient in French or German but did not use languages other than English on a daily basis and did not fulfil the definition of bilingualism used in the study. All of the bilingual group were UK born (Wales 45, England 1). Nearly all of the monolingual group were born in the UK (Wales 13, England 39, and Scotland 2) with three immigrants from English speaking communities (one from each of Australia, South Africa, and Ireland). For baseline categorical variables the only significant difference after correction was in education level (monolingual > bilingual). Education was therefore included as a factor in subsequent analyses. There were no significant differences in the stage or motor severity of PD nor were there differences in LED or cholinergic load; see Table 1.

### 3.2. Language Tests

Monolinguals performed significantly better than bilinguals with medium to large positive effect sizes for the NART-R and BPVS; see Tables 2 and 3.

### 3.3. Comparison of Monolinguals and Bilinguals on Performance of EF Tests

Sixty-six of the cohort completed the full set of tests used in the two-factor ANOVA (full data: monolingual 42 and bilingual 24; incomplete data: monolingual 15 and bilingual 22 with 6 excluded since they reverted to Welsh during completion of the verbal and category fluency tests); see Table 2. There were no significant differences between bilinguals and monolinguals on measures of EF. In order to improve the numbers of bilinguals in the analysis a second two-factor ANOVA was performed using seventy-two of the cohort who completed the tests excluding verbal and category fluency (full data: monolingual 42 and bilingual 30; incomplete data: monolingual 15 and bilingual 16), but this made no difference to the overall results (significantly better performance for monolinguals for NART-R *P* = .001 and BPVRS *P* = .004 but all results for executive tests nonsignificant). Confidence intervals for all effect sizes were wide and included negative effects. In order to further account for any possible effects of education those with a total number of 16 or more years of education were excluded from the analysis (total cohort excludes 13 from monolingual group and 3 from bilingual group but in the ANOVA analysis it excluded 9 from the monolingual group and 2 from bilingual group). A *t*-test was then performed on the two language groups which showed no significant difference in years of education between monolinguals and bilinguals (*P* = .136) and a further ANOVA showed persistent effects on NART-R (*P* = .002) and BPVRS (*P* = .006) but no significant effects on performance on tests of executive function. Due to the possibility that the results for the NART-R as a measure of intelligence were confounding any effect of bilingualism in addition to the effect of education [19, 57], a secondary analysis for the whole cohort was performed to include the NART-R as a covariate. This analysis still confirmed no effect of bilingualism on any measure of EF (all *P* > .05).

### 3.4. The Effects of Different Degrees of Language Use in Bilinguals on Performance of EF Tests

Comparisons between the two clusters of bilinguals are summarised in Table 3. There was a significant difference in performance on the NART-R favouring those in Cluster 1 (less frequent Welsh speakers). There were no significant differences on other language tests. On tests of EF there were no significant differences between the two clusters after correction for multiple comparisons.

There was a correlation between the bilingual index (higher score = higher degree of bilingualism) and better performance on one test for nonverbal reasoning (RCPM *ρ* = .38, *P* = .01) and one test of working memory (Keep track task *ρ* = .34, *P* = .02). There was no significant correlation between the bilingualism index and performance of other tests of EF.

## 4. Discussion

There was no significant difference between monolinguals and bilinguals in the performance of EF tests. These findings overall do not confirm the first hypothesis that bilinguals with PD will show better performance on executive control tasks than monolinguals.

A higher degree of bilingualism did correlate with better performance only on one test of nonverbal general reasoning (RCPM) and one test of working memory (Keep Track task) but not on any other test of executive function. Welsh/English bilinguals who used Welsh less in daily life had better performance on one test of vocabulary but not on tests of EF. Overall, however, the results tend not to confirm the second hypothesis that bilinguals with PD who have a higher degree of bilingualism will show better performance on executive control tasks than those with a lower degree of bilingualism.

This present study is the first study to examine the effects of bilingualism as a form of cognitive reserve on EF in PD. The reasons for the negative effects in the study need to be explored in order to understand why the bilingual advantage is not always found and to understand the role of EF [30]. EF was studied since it was proposed that the mechanism by which bilingualism provides cognitive reserve is through compensation of more intact EF processes. The finding of no effect of bilingualism on EF in PD conflicts with some previous findings in AD. A study of EF in AD published following the commencement of our study confirmed a delayed presentation with AD in bilinguals, and although bilinguals and monolinguals had comparable EF the bilinguals were older and likely to have a greater degree of brain pathology [58]. In our study in PD there was no difference in age or disease severity between the monolinguals and bilinguals. It is possible that when the EF system is impaired, as in many people with PD, there may be no resources for compensation and no evidence of reserve, with PD producing similar results to those seen in multidomain amnestic MCI [31]. The effects of cognitive reserve may be difficult to demonstrate in PD since it is a complex heterogeneous condition presenting primarily as a motor disorder with the subsequent development of cognitive impairment. The fact that the rate of cognitive decline in PD may be nonlinear may have confounded the results. A previous longitudinal study using the MMSE has shown that initially there is a relatively stable period of cognitive function in PD in which there is a shallow rate of decline, followed by an inflection point, the timing of which may be very variable, when the decline gains momentum towards dementia [59]. As suggested in the discussion of our previous meta-analysis [18] higher cognitive reserve may theoretically slow the initial decline and delay the inflection point, following which pathology in advanced PD, driven by ageing [7], overwhelms any beneficial effect of cognitive reserve on cognitive decline. Future studies of cognitive reserve in PD should therefore include longitudinal analysis of the rate of cognitive decline and development of dementia.

The nature of the bilingual pairing of Welsh/English may affect the nature of the interaction with neuropsychological function and language as it has done in other language pairs [27]. This is the first study of cognitive reserve in any neurodegenerative condition in Welsh/English bilinguals and the effect has not yet been demonstrated in this population for AD. Another important consideration is the degree of bilingualism, which may be important in considering the effects of cognitive reserve [26]. In this study there was a significant correlation between the degree of bilingualism and one test of nonverbal general reasoning and one of working memory although there was no correlation with any other test of executive function. The effects of the degree of bilingualism may warrant further study since the nature of the different bilingual pairs and degree of switching between the languages may influence the effect of cognitive reserve [27]. In the present study there was no effect of a greater daily use of Welsh on EF although there was an effect on a test of vocabulary favouring those who used less Welsh in daily life. It may be that the amount of daily language switching in our Welsh/English group is less than that in other bilingual groups.

The overall pattern of results showed that monolinguals had higher performance on language tests except the BNT. In English and Welsh versions of the BNT many words are very similar and this may have reduced the expected monolingual advantage on the test. The differences in language performance in the study confirm the findings of previous studies in healthy older adults demonstrating lifelong differences in vocabulary between monolinguals and bilinguals, with bilinguals typically achieving poorer scores on vocabulary, naming, and fluency tasks [30, 60, 61].

The potential limitations of the study must be considered in interpreting the results. The study was performed in people with relatively early PD and the effects of long term cognitive reserve in more advanced PD are unclear and require further study. The potential effects of cognitive reserve may have been masked by the differences in education between our ML and BL groups, although we did include educational level as a fixed factor in the analysis to try to correct for these differences. We also performed a further analysis to exclude those with very high levels of education (more in the monolingual group) but there were still no significant results for performance on executive tasks. In order to take into consideration the contribution of intelligence [19, 57] an additional analysis using the NART-R as a covariate still did not confirm any added benefit for bilingualism. There may, however, have been other cognitive and social factors not identified in the study which made the groups differ in a way which influenced the results. In comparing the daily use of both languages, the cluster using Welsh most of the time was small in number, which affected the significance of the results. Due to the language environment in Wales where most signs and many documents are bilingual, monolinguals had some exposure to Welsh with 21% having been taught basic Welsh in school or adulthood (although only one participant used Welsh up to 50% of the time on a daily basis). Immigration can be a confounding factor in many studies of bilingualism and may provide additional enrichment to lifestyle enhancing cognitive reserve [62]. Although many of the monolingual group in this study had moved within the UK only three were actual immigrants to the UK as a whole from English speaking communities. It is, however, possible that there may be an additional enriching effect of immigration to one area from another area within a country which confounds the results in this population.

## 5. Conclusions

In a study of Welsh/English bilingualism in PD monolinguals showed an advantage in performance of English language tests. There were no differences in performance of EF tests between monolinguals and bilinguals. Those who used Welsh less in daily life had better performance on one test of vocabulary. Although the degree of bilingualism showed a correlation with individual tests of nonverbal general reasoning and working memory there was no overall effect on EF. The reasons why the expected benefit in EF in Welsh-English bilinguals with PD was not found require further study. Future studies in PD should include other language pairs, analysis of the effects of the degree of bilingualism, and longitudinal analysis of cognitive decline or dementia together with structural or functional neuroimaging.

## Figures and Tables

**(a):** 

Variable-mean value (SD)	Monolingual	Bilingual	Chi squared	Significance	ANOVA *F*	Significance
	*N* = 57	*N* = 46				
Age	71.5 (7.9)	71.3 (10.5)			*0.01 *	.89
Age at diagnosis	64.6 (9.2)	65.2 (13.3)			*0.07 *	.79
Education years	13.0 (2.8)	11.8 (2.3)			*5.26 *	.02
UPDRS motor	20.4 (8.8)	23.1 (12.2)			*1.49 *	.22
HADS depression	3.9 (2.4)	4.3 (3.1)			*0.59 *	.44
HADS anxiety	4.7 (3.1)	5.3 (3.7)			*0.78 *	.37
MMSE	28.6 (2.0)	27.9 (2.4)			*3.04 *	.08
LED	576 (387)	493 (386)			*1.171 *	.28
Anticholinergic cognitive burden	0.47 (1.3)	0.82 (1.3)			*1.74 *	.18
Male	40	31	0.09	.46
Female	17	15		

**(b):** 

Variable	Category	Monolingual	Bilingual	Chi squared	Significance	ANOVA *F*	Significance
		*N* = 57	*N* = 46				
Socioeconomic status^∗^	(I) Professional	7	1	5.53	.23		
	(II) Managerial/technical	21	17				
	(III N) nonmanual skilled	9	8				
	(III M) manual skilled	14	10				
	(IV) Partly skilled	6	10				
	(V) Unskilled	2	0				

Education years category	(1) 7–11	20	23	5.02	.08		
	(2) 12–15	23	19				
	(3) 16–20	14	4				

Education level category	(1) Left before 16, no qualification	9	22	**16.38**	**.001**		
	(2) Secondary only	9	10				
	(3) Further education/vocational	29	11				
	(4) University & higher degrees	10	3				

Hoehn and Yahr [20]	1	31	32	2.19	.33		
	2	19	11				
	3 & 4	6	3				

^∗^Classification based on occupation (64).

Hospital Anxiety and Depression Scale (HADS), Mini-Mental State Examination (MMSE), Unified Parkinson's Disease Rating Scale (UPDRS), and levodopa equivalent dose (LED).

**Table 2:** Comparison of monolinguals and bilinguals on language tests, EF tests.

Variables	Effects of language: monolingual (ML) and bilingual (BL)				
	After education level as fixed factor in 2-factor ANOVA				
	Monolingual mean (SD) *N* = 42	Bilingual mean (SD) *N* = 24	Direction (see footnote)	Effect size (95% confidence intervals)	Significance *P* value
Language tests					
NART-R errors	**11.29 (8.84)**	**20.46 (8.88)**	**ML** > **BL**	**.83 (0.31, 1.35)**	**.003**
British Picture Vocabulary Scale total correct	**57.31 (2.62)**	**54.08 (5.04)**	**ML** > **BL**	**.69 (0.18, 1.20)**	**.013**
Boston Naming Test English total	14.50 (0.80)	14.37 (0.77)	ML > BL	.01 (−0.48, 0.51)	.95
EF, attention variables					
D-KEFS verbal fluency total correct raw score	42.69 (13.73)	40.29 (10.74)	ML > BL	.18 (−0.32, 0.68)	.50
D-KEFS category fluency total correct raw score	37.36 (11.41)	38.71 (9.44)	BL > ML	.20 (−0.30, 0.80)	.45
D-KEFS design fluency filled + empty + switching total correct raw score	23.69 (7.14)	22.63 (8.55)	ML > BL	.02 (−0.44, 0.52)	.93
D-KEFS TMT Part 4 raw score (seconds)	130.86 (58.40)	144.67 (70.74)	ML > BL	.19 (−0.31, 0.69)	.65
RCPM total correct	31.69 (4.24)	31.54 (4.24)	ML > BL	.12 (−0.38, 0.62)	.64
Digit Span backwards total	6.69 (2.29)	6.79 (2.12)	BL > ML	.17 (−0.33, 0.67)	.52
Spatial Span backwards total	6.55 (1.96)	6.17 (1.60)	ML > BL	.07 (−0.43, 0.57)	.77
TEA Elevator Counting raw score	6.90 (.37)	6.92 (.28)	ML > BL	.03 (−0.47, 0.53)	.89
TEA Elevator Counting with distraction raw score	7.48 (2.49)	6.71 (2.86)	ML > BL	.17 (−0.33, 0.67)	.51
Keep Track task total correct	7.79 (2.20)	7.67 (2.12)	ML > BL	.02 (−0.48, 0.52)	.92
Simon task mean reaction time difference (ms)	83 (294)	129 (272)	ML > BL	.13 (−0.37, 0.63)	.61
Stroop reaction time difference (incongruent − congruent reaction time) (ms)	1011 (746)	1311 (699)	ML > BL	.41 (−0.09, 0.91)	.12
Go No-Go commission errors %	5.01 (8.75)	4.60 (7.33)	BL > ML	.03 (−0.47, 0.53)	.90
Go No-Go mean reaction time (ms)	509 (94)	512 (112)	ML > BL	.01 (−0.49, 0.51)	.95

Note: direction indicates which group scored better monolingual (ML) versus bilingual (BL). Effect size (SMD) was calculated as the difference between the estimated marginal means for ML and BL groups divided by the square root of the error mean square term. Significant results in **bold** after adjustment for multiple comparisons using Holm-Bonferroni method separately for descriptive variables and combined EF/attention/function variables.

Delis-Kaplan Executive Function System (D-KEFS), National Adult Reading Test-Revised (NART-R), Raven's Coloured Progressive Matrices (RCPM), Tests of Everyday Activity (TEA), and Trail Making Test (TMT).

**Table 3:** The effects of different degrees of language use in bilinguals.

Variables	Degree of bilingualism-cluster analysis ANOVA Cluster 1: less frequent Welsh speakers *n* = 18 Cluster 2: more frequent Welsh speakers *n* = 27			Bilingualism index Nonparametric correlations	
	df	*F*	Significance *P* value	Spearman's rho *n* = 46	Significance *P* value
Language tests					
NART-R errors	**40**	***7.31***	**.010**	0.001	.99
British Picture Vocabulary Scale total correct	43	*5.12 *	.029	−0.07	.60
Boston Naming Test English total	43	*2.77 *	.10	0.02	.85
EF, attention variables					
D-KEFS verbal fluency total correct raw score	42	*3.36 *	.07	0.03	.76
D-KEFS category fluency total correct raw score	41	*0.48 *	.48	0.01	.93
D-KEFS design fluency filled + empty + switching total correct raw score	40	*0.24 *	.62	0.06	.66
D-KEFS TMT Part 4 raw score (seconds)	33	*0.01 *	.91	−0.23	.15
RCPM total correct	40	*1.4 *	.24	**0.38**	**.01**
Digit Span backwards total	42	*0.35 *	.55	0.19	.20
Spatial Span backwards total	41	*0.64 *	0.42	0.03	.81
TEA Elevator Counting raw score	37	*0.002 *	0.96	0.17	.26
TEA Elevator Counting with distraction raw score	36	*0.02 *	0.87	0.12	.46
Keep Track task total correct	36	*0.09 *	0.75	**0.34**	**.02**
Simon task mean reaction time difference (ms)	41	*0.75 *	0.38	−0.11	.45
Stroop reaction time difference (incongruent − congruent reaction time) (ms)	35	*0.83 *	0.36	−0.10	.54
Go No-Go commission errors %	39	*3.66 *	0.06	−0.06	.69
Go No-Go mean reaction time (ms)	39	*0.53 *	0.46	−0.18	.24

Significant results in **bold** after adjustment for multiple comparisons using Holm-Bonferroni method separately for descriptive variables and combined EF/attention/function variables in ANOVA.

Delis-Kaplan Executive Function System (D-KEFS), National Adult Reading Test-Revised (NART-R), Raven's Coloured Progressive Matrices (RCPM), Tests of Everyday Activity (TEA), and Trail Making Test (TMT).
